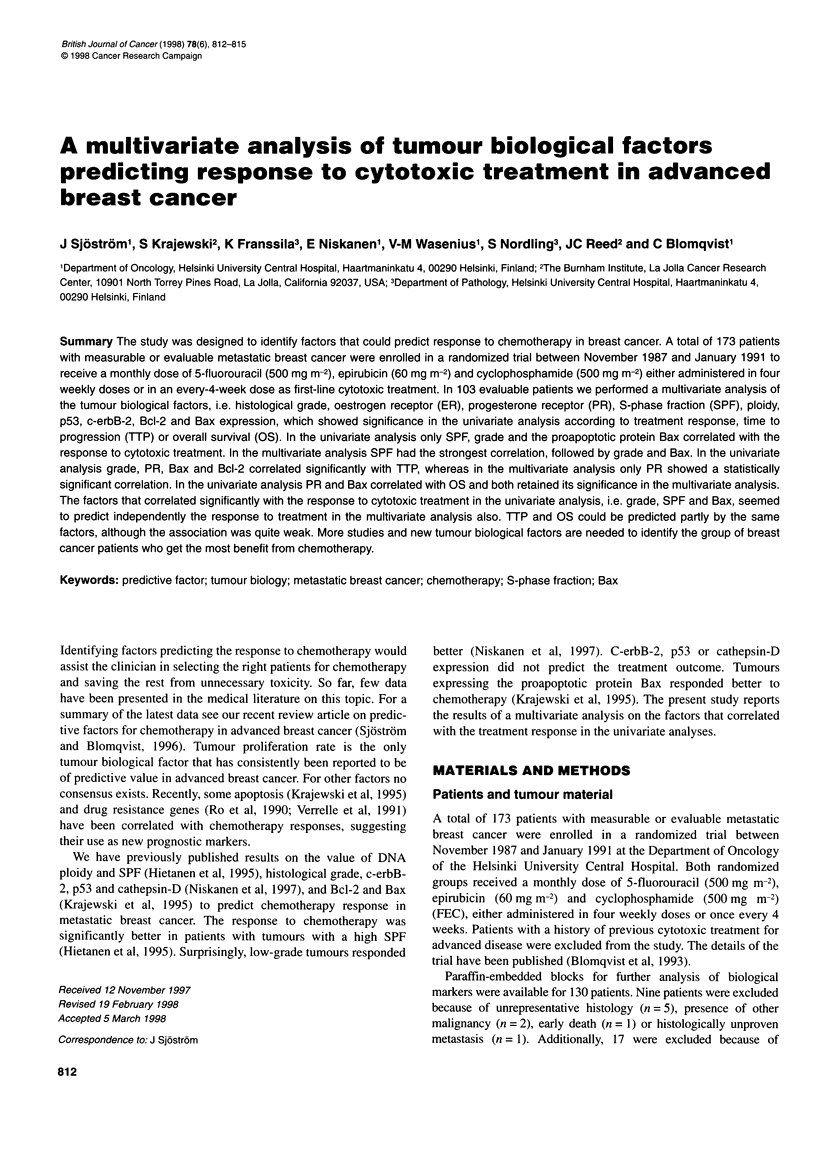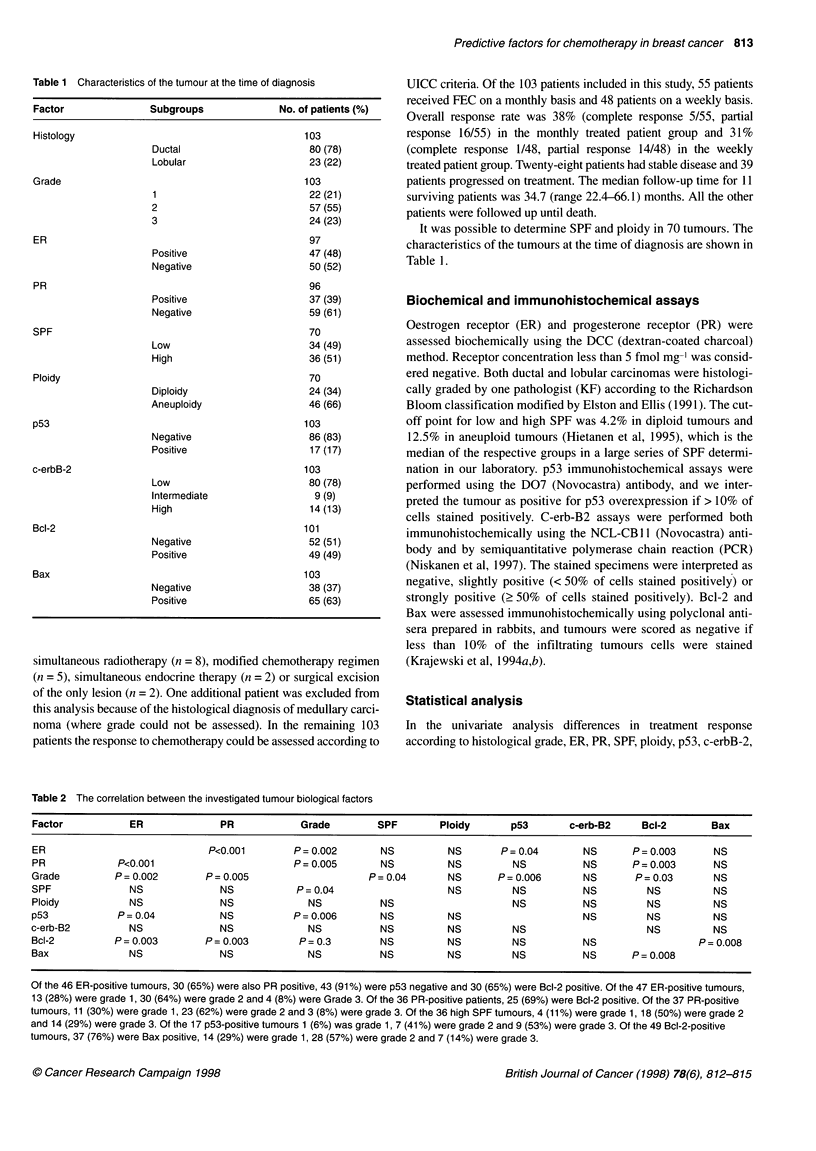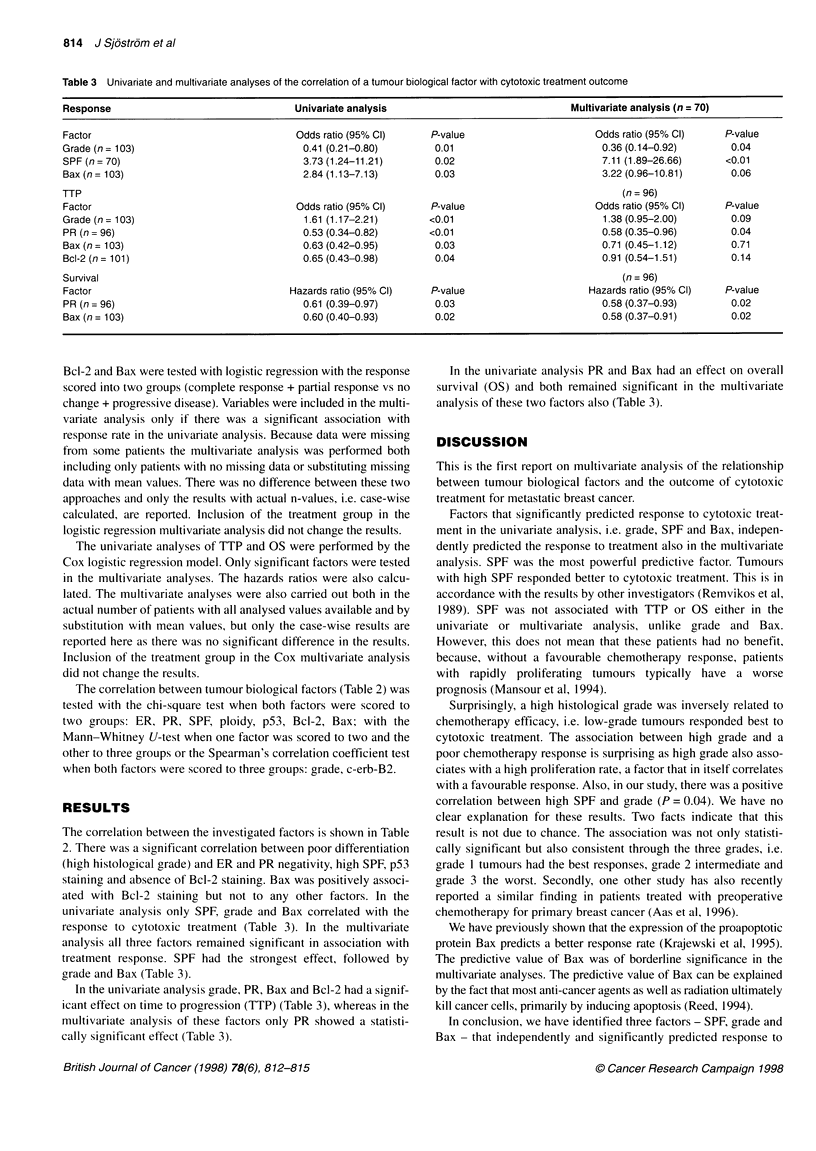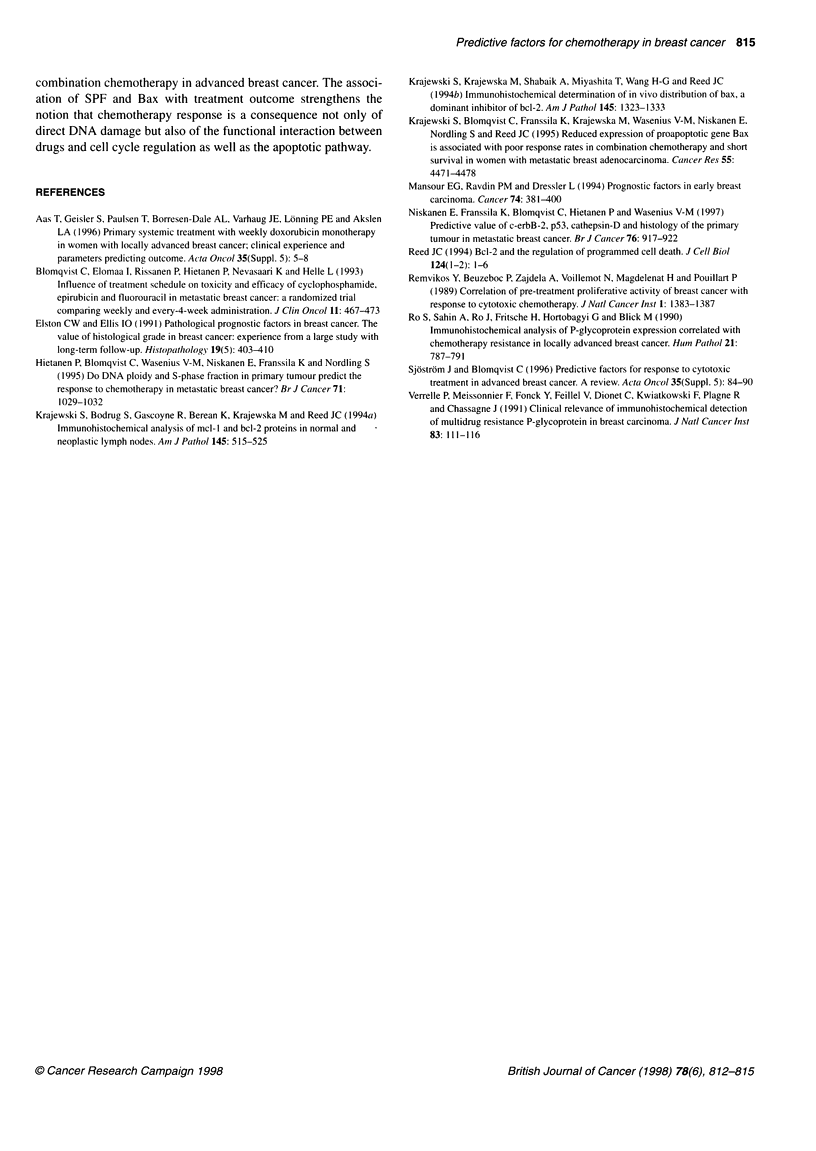# A multivariate analysis of tumour biological factors predicting response to cytotoxic treatment in advanced breast cancer.

**DOI:** 10.1038/bjc.1998.584

**Published:** 1998-09

**Authors:** J. Sjöström, S. Krajewski, K. Franssila, E. Niskanen, V. M. Wasenius, S. Nordling, J. C. Reed, C. Blomqvist

**Affiliations:** Department of Oncology, Helsinki University Central Hospital, Finland.

## Abstract

The study was designed to identify factors that could predict response to chemotherapy in breast cancer. A total of 173 patients with measurable or evaluable metastatic breast cancer were enrolled in a randomized trial between November 1987 and January 1991 to receive a monthly dose of 5-fluorouracil (500 mg m(-2)), epirubicin (60 mg m(-2)) and cyclophosphamide (500 mg m(-2)) either administered in four weekly doses or in an every-4-week dose as first-line cytotoxic treatment. In 103 evaluable patients we performed a multivariate analysis of the tumour biological factors, i.e. histological grade, oestrogen receptor (ER), progesterone receptor (PR), S-phase fraction (SPF), ploidy, p53, c-erbB-2, Bcl-2 and Bax expression, which showed significance in the univariate analysis according to treatment response, time to progression (TTP) or overall survival (OS). In the univariate analysis only SPF, grade and the proapoptotic protein Bax correlated with the response to cytotoxic treatment. In the multivariate analysis SPF had the strongest correlation, followed by grade and Bax. In the univariate analysis grade, PR, Bax and Bcl-2 correlated significantly with TTP, whereas in the multivariate analysis only PR showed a statistically significant correlation. In the univariate analysis PR and Bax correlated with OS and both retained its significance in the multivariate analysis. The factors that correlated significantly with the response to cytotoxic treatment in the univariate analysis, i.e. grade, SPF and Bax, seemed to predict independently the response to treatment in the multivariate analysis also. TTP and OS could be predicted partly by the same factors, although the association was quite weak. More studies and new tumour biological factors are needed to identify the group of breast cancer patients who get the most benefit from chemotherapy.


British Journal of Cancer (1998) 78(6), 812–815
© 1998 Cancer Research Campaign

### A multivariate analysis of tumour biological factors predicting response to cytotoxic treatment in advanced breast cancer

**J Sjöström[1], S Krajewski[2], K Franssila[3], E Niskanen[1], V-M Wasenius[1], S Nordling[3], JC Reed[2] and C Blomqvist[1]**

[1]Department of Oncology, Helsinki University Central Hospital, Haartmaninkatu 4, 00290 Helsinki, Finland; [2]The Burnham Institute, La Jolla Cancer Research Center, 10901 North Torrey Pines Road, La Jolla, California 92037, USA; [3]Department of Pathology, Helsinki University Central Hospital, Haartmaninkatu 4, 00290 Helsinki, Finland

**Summary** The study was designed to identify factors that could predict response to chemotherapy in breast cancer. A total of 173 patients with measurable or evaluable metastatic breast cancer were enrolled in a randomized trial between November 1987 and January 1991 to receive a monthly dose of 5-fluorouracil (500 mg $m^{-2}$), epirubicin (60 mg $m^{-2}$) and cyclophosphamide (500 mg $m^{-2}$) either administered in four weekly doses or in an every-4-week dose as first-line cytotoxic treatment. In 103 evaluable patients we performed a multivariate analysis of the tumour biological factors, i.e. histological grade, oestrogen receptor (ER), progesterone receptor (PR), S-phase fraction (SPF), ploidy, p53, c-erbB-2, Bcl-2 and Bax expression, which showed significance in the univariate analysis according to treatment response, time to progression (TTP) or overall survival (OS). In the univariate analysis only SPF, grade and the proapoptotic protein Bax correlated with the response to cytotoxic treatment. In the multivariate analysis SPF had the strongest correlation, followed by grade and Bax. In the univariate analysis grade, PR, Bax and Bcl-2 correlated significantly with TTP, whereas in the multivariate analysis only PR showed a statistically significant correlation. In the univariate analysis PR and Bax correlated with OS and both retained its significance in the multivariate analysis. The factors that correlated significantly with the response to cytotoxic treatment in the univariate analysis, i.e. grade, SPF and Bax, seemed to predict independently the response to treatment in the multivariate analysis also. TTP and OS could be predicted partly by the same factors, although the association was quite weak. More studies and new tumour biological factors are needed to identify the group of breast cancer patients who get the most benefit from chemotherapy.

**Keywords:** predictive factor; tumour biology; metastatic breast cancer; chemotherapy; S-phase fraction; Bax

Identifying factors predicting the response to chemotherapy would assist the clinician in selecting the right patients for chemotherapy and saving the rest from unnecessary toxicity. So far, few data have been presented in the medical literature on this topic. For a summary of the latest data see our recent review article on predictive factors for chemotherapy in advanced breast cancer (Sjöström and Blomqvist, 1996). Tumour proliferation rate is the only tumour biological factor that has consistently been reported to be of predictive value in advanced breast cancer. For other factors no consensus exists. Recently, some apoptosis (Krajewski et al, 1995) and drug resistance genes (Ro et al, 1990; Verrelle et al, 1991) have been correlated with chemotherapy responses, suggesting their use as new prognostic markers.

We have previously published results on the value of DNA ploidy and SPF (Hietanen et al, 1995), histological grade, c-erbB-2, p53 and cathepsin-D (Niskanen et al, 1997), and Bcl-2 and Bax (Krajewski et al, 1995) to predict chemotherapy response in metastatic breast cancer. The response to chemotherapy was significantly better in patients with tumours with a high SPF (Hietanen et al, 1995). Surprisingly, low-grade tumours responded better (Niskanen et al, 1997). C-erbB-2, p53 or cathepsin-D expression did not predict the treatment outcome. Tumours expressing the proapoptotic protein Bax responded better to chemotherapy (Krajewski et al, 1995). The present study reports the results of a multivariate analysis on the factors that correlated with the treatment response in the univariate analyses.

*Received 12 November 1997*
*Revised 19 February 1998*
*Accepted 5 March 1998*

*Correspondence to:* J Sjöström

### MATERIALS AND METHODS

### Patients and tumour material

A total of 173 patients with measurable or evaluable metastatic breast cancer were enrolled in a randomized trial between November 1987 and January 1991 at the Department of Oncology of the Helsinki University Central Hospital. Both randomized groups received a monthly dose of 5-fluorouracil (500 mg $m^{-2}$), epirubicin (60 mg $m^{-2}$) and cyclophosphamide (500 mg $m^{-2}$) (FEC), either administered in four weekly doses or once every 4 weeks. Patients with a history of previous cytotoxic treatment for advanced disease were excluded from the study. The details of the trial have been published (Blomqvist et al, 1993).

Paraffin-embedded blocks for further analysis of biological markers were available for 130 patients. Nine patients were excluded because of unrepresentative histology ($n = 5$), presence of other malignancy ($n = 2$), early death ($n = 1$) or histologically unproven metastasis ($n = 1$). Additionally, 17 were excluded because of

**Table 1** Characteristics of the tumour at the time of diagnosis

| Factor | Subgroups | No. of patients (%) |
|---|---|---|
| Histology | | 103 |
| | Ductal | 80 (78) |
| | Lobular | 23 (22) |
| Grade | | 103 |
| | 1 | 22 (21) |
| | 2 | 57 (55) |
| | 3 | 24 (23) |
| ER | | 97 |
| | Positive | 47 (48) |
| | Negative | 50 (52) |
| PR | | 96 |
| | Positive | 37 (39) |
| | Negative | 59 (61) |
| SPF | | 70 |
| | Low | 34 (49) |
| | High | 36 (51) |
| Ploidy | | 70 |
| | Diploidy | 24 (34) |
| | Aneuploidy | 46 (66) |
| p53 | | 103 |
| | Negative | 86 (83) |
| | Positive | 17 (17) |
| c-erbB-2 | | 103 |
| | Low | 80 (78) |
| | Intermediate | 9 (9) |
| | High | 14 (13) |
| Bcl-2 | | 101 |
| | Negative | 52 (51) |
| | Positive | 49 (49) |
| Bax | | 103 |
| | Negative | 38 (37) |
| | Positive | 65 (63) |

simultaneous radiotherapy ($n = 8$), modified chemotherapy regimen ($n = 5$), simultaneous endocrine therapy ($n = 2$) or surgical excision of the only lesion ($n = 2$). One additional patient was excluded from this analysis because of the histological diagnosis of medullary carcinoma (where grade could not be assessed). In the remaining 103 patients the response to chemotherapy could be assessed according to UICC criteria. Of the 103 patients included in this study, 55 patients received FEC on a monthly basis and 48 patients on a weekly basis. Overall response rate was 38% (complete response 5/55, partial response 16/55) in the monthly treated patient group and 31% (complete response 1/48, partial response 14/48) in the weekly treated patient group. Twenty-eight patients had stable disease and 39 patients progressed on treatment. The median follow-up time for 11 surviving patients was 34.7 (range 22.4–66.1) months. All the other patients were followed up until death.

It was possible to determine SPF and ploidy in 70 tumours. The characteristics of the tumours at the time of diagnosis are shown in Table 1.

### Biochemical and immunohistochemical assays

Oestrogen receptor (ER) and progesterone receptor (PR) were assessed biochemically using the DCC (dextran-coated charcoal) method. Receptor concentration less than 5 fmol $mg^{-1}$ was considered negative. Both ductal and lobular carcinomas were histologically graded by one pathologist (KF) according to the Richardson Bloom classification modified by Elston and Ellis (1991). The cut-off point for low and high SPF was 4.2% in diploid tumours and 12.5% in aneuploid tumours (Hietanen et al, 1995), which is the median of the respective groups in a large series of SPF determination in our laboratory. p53 immunohistochemical assays were performed using the DO7 (Novocastra) antibody, and we interpreted the tumour as positive for p53 overexpression if > 10% of cells stained positively. C-erb-B2 assays were performed both immunohistochemically using the NCL-CB11 (Novocastra) antibody and by semiquantitative polymerase chain reaction (PCR) (Niskanen et al, 1997). The stained specimens were interpreted as negative, slightly positive (< 50% of cells stained positively) or strongly positive (≥ 50% of cells stained positively). Bcl-2 and Bax were assessed immunohistochemically using polyclonal antisera prepared in rabbits, and tumours were scored as negative if less than 10% of the infiltrating tumours cells were stained (Krajewski et al, 1994*a*,*b*).

### Statistical analysis

In the univariate analysis differences in treatment response according to histological grade, ER, PR, SPF, ploidy, p53, c-erbB-2,

**Table 2** The correlation between the investigated tumour biological factors

| Factor | ER | PR | Grade | SPF | Ploidy | p53 | c-erb-B2 | Bcl-2 | Bax |
|---|---|---|---|---|---|---|---|---|---|
| ER | | $P$<0.001 | $P$ = 0.002 | NS | NS | $P$ = 0.04 | NS | $P$ = 0.003 | NS |
| PR | $P$<0.001 | | $P$ = 0.005 | NS | NS | NS | NS | $P$ = 0.003 | NS |
| Grade | $P$ = 0.002 | $P$ = 0.005 | | $P$ = 0.04 | NS | $P$ = 0.006 | NS | $P$ = 0.03 | NS |
| SPF | NS | NS | $P$ = 0.04 | | NS | NS | NS | NS | NS |
| Ploidy | NS | NS | NS | NS | | NS | NS | NS | NS |
| p53 | $P$ = 0.04 | NS | $P$ = 0.006 | NS | NS | | NS | NS | NS |
| c-erb-B2 | NS | NS | NS | NS | NS | NS | | NS | NS |
| Bcl-2 | $P$ = 0.003 | $P$ = 0.003 | $P$ = 0.3 | NS | NS | NS | NS | | $P$ = 0.008 |
| Bax | NS | NS | NS | NS | NS | NS | NS | $P$ = 0.008 | |

Of the 46 ER-positive tumours, 30 (65%) were also PR positive, 43 (91%) were p53 negative and 30 (65%) were Bcl-2 positive. Of the 47 ER-positive tumours, 13 (28%) were grade 1, 30 (64%) were grade 2 and 4 (8%) were Grade 3. Of the 36 PR-positive patients, 25 (69%) were Bcl-2 positive. Of the 37 PR-positive tumours, 11 (30%) were grade 1, 23 (62%) were grade 2 and 3 (8%) were grade 3. Of the 36 high SPF tumours, 4 (11%) were grade 1, 18 (50%) were grade 2 and 14 (29%) were grade 3. Of the 17 p53-positive tumours 1 (6%) was grade 1, 7 (41%) were grade 2 and 9 (53%) were grade 3. Of the 49 Bcl-2-positive tumours, 37 (76%) were Bax positive, 14 (29%) were grade 1, 28 (57%) were grade 2 and 7 (14%) were grade 3.

© *Cancer Research Campaign 1998*

Table 3 Univariate and multivariate analyses of the correlation of a tumour biological factor with cytotoxic treatment outcome

| Response | Univariate analysis | | Multivariate analysis ($n$ = 70) | |
|---|---|---|---|---|
| Factor | Odds ratio (95% CI) | $P$-value | Odds ratio (95% CI) | $P$-value |
| Grade ($n$ = 103) | 0.41 (0.21–0.80) | 0.01 | 0.36 (0.14–0.92) | 0.04 |
| SPF ($n$ = 70) | 3.73 (1.24–11.21) | 0.02 | 7.11 (1.89–26.66) | <0.01 |
| Bax ($n$ = 103) | 2.84 (1.13–7.13) | 0.03 | 3.22 (0.96–10.81) | 0.06 |
| TTP | | | ($n$ = 96) | |
| Factor | Odds ratio (95% CI) | $P$-value | Odds ratio (95% CI) | $P$-value |
| Grade ($n$ = 103) | 1.61 (1.17–2.21) | <0.01 | 1.38 (0.95–2.00) | 0.09 |
| PR ($n$ = 96) | 0.53 (0.34–0.82) | <0.01 | 0.58 (0.35–0.96) | 0.04 |
| Bax ($n$ = 103) | 0.63 (0.42–0.95) | 0.03 | 0.71 (0.45–1.12) | 0.71 |
| Bcl-2 ($n$ = 101) | 0.65 (0.43–0.98) | 0.04 | 0.91 (0.54–1.51) | 0.14 |
| Survival | | | ($n$ = 96) | |
| Factor | Hazards ratio (95% CI) | $P$-value | Hazards ratio (95% CI) | $P$-value |
| PR ($n$ = 96) | 0.61 (0.39–0.97) | 0.03 | 0.58 (0.37–0.93) | 0.02 |
| Bax ($n$ = 103) | 0.60 (0.40–0.93) | 0.02 | 0.58 (0.37–0.91) | 0.02 |

Bcl-2 and Bax were tested with logistic regression with the response scored into two groups (complete response + partial response vs no change + progressive disease). Variables were included in the multivariate analysis only if there was a significant association with response rate in the univariate analysis. Because data were missing from some patients the multivariate analysis was performed both including only patients with no missing data or substituting missing data with mean values. There was no difference between these two approaches and only the results with actual n-values, i.e. case-wise calculated, are reported. Inclusion of the treatment group in the logistic regression multivariate analysis did not change the results.

The univariate analyses of TTP and OS were performed by the Cox logistic regression model. Only significant factors were tested in the multivariate analyses. The hazards ratios were also calculated. The multivariate analyses were also carried out both in the actual number of patients with all analysed values available and by substitution with mean values, but only the case-wise results are reported here as there was no significant difference in the results. Inclusion of the treatment group in the Cox multivariate analysis did not change the results.

The correlation between tumour biological factors (Table 2) was tested with the chi-square test when both factors were scored to two groups: ER, PR, SPF, ploidy, p53, Bcl-2, Bax; with the Mann–Whitney $U$-test when one factor was scored to two and the other to three groups or the Spearman's correlation coefficient test when both factors were scored to three groups: grade, c-erb-B2.

### RESULTS

The correlation between the investigated factors is shown in Table 2. There was a significant correlation between poor differentiation (high histological grade) and ER and PR negativity, high SPF, p53 staining and absence of Bcl-2 staining. Bax was positively associated with Bcl-2 staining but not to any other factors. In the univariate analysis only SPF, grade and Bax correlated with the response to cytotoxic treatment (Table 3). In the multivariate analysis all three factors remained significant in association with treatment response. SPF had the strongest effect, followed by grade and Bax (Table 3).

In the univariate analysis grade, PR, Bax and Bcl-2 had a significant effect on time to progression (TTP) (Table 3), whereas in the multivariate analysis of these factors only PR showed a statistically significant effect (Table 3).

In the univariate analysis PR and Bax had an effect on overall survival (OS) and both remained significant in the multivariate analysis of these two factors also (Table 3).

### DISCUSSION

This is the first report on multivariate analysis of the relationship between tumour biological factors and the outcome of cytotoxic treatment for metastatic breast cancer.

Factors that significantly predicted response to cytotoxic treatment in the univariate analysis, i.e. grade, SPF and Bax, independently predicted the response to treatment also in the multivariate analysis. SPF was the most powerful predictive factor. Tumours with high SPF responded better to cytotoxic treatment. This is in accordance with the results by other investigators (Remvikos et al, 1989). SPF was not associated with TTP or OS either in the univariate or multivariate analysis, unlike grade and Bax. However, this does not mean that these patients had no benefit, because, without a favourable chemotherapy response, patients with rapidly proliferating tumours typically have a worse prognosis (Mansour et al, 1994).

Surprisingly, a high histological grade was inversely related to chemotherapy efficacy, i.e. low-grade tumours responded best to cytotoxic treatment. The association between high grade and a poor chemotherapy response is surprising as high grade also associates with a high proliferation rate, a factor that in itself correlates with a favourable response. Also, in our study, there was a positive correlation between high SPF and grade ($P$ = 0.04). We have no clear explanation for these results. Two facts indicate that this result is not due to chance. The association was not only statistically significant but also consistent through the three grades, i.e. grade 1 tumours had the best responses, grade 2 intermediate and grade 3 the worst. Secondly, one other study has also recently reported a similar finding in patients treated with preoperative chemotherapy for primary breast cancer (Aas et al, 1996).

We have previously shown that the expression of the proapoptotic protein Bax predicts a better response rate (Krajewski et al, 1995). The predictive value of Bax was of borderline significance in the multivariate analyses. The predictive value of Bax can be explained by the fact that most anti-cancer agents as well as radiation ultimately kill cancer cells, primarily by inducing apoptosis (Reed, 1994).

In conclusion, we have identified three factors – SPF, grade and Bax – that independently and significantly predicted response to

 © Cancer Research Campaign 1998

combination chemotherapy in advanced breast cancer. The association of SPF and Bax with treatment outcome strengthens the notion that chemotherapy response is a consequence not only of direct DNA damage but also of the functional interaction between drugs and cell cycle regulation as well as the apoptotic pathway.

### REFERENCES

Aas T, Geisler S, Paulsen T, Borresen-Dale AL, Varhaug JE, Lönning PE and Akslen LA (1996) Primary systemic treatment with weekly doxorubicin monotherapy in women with locally advanced breast cancer; clinical experience and parameters predicting outcome. *Acta Oncol* **35**(Suppl. 5): 5–8

Blomqvist C, Elomaa I, Rissanen P, Hietanen P, Nevasaari K and Helle L (1993) Influence of treatment schedule on toxicity and efficacy of cyclophosphamide, epirubicin and fluorouracil in metastatic breast cancer: a randomized trial comparing weekly and every-4-week administration. *J Clin Oncol* **11**: 467–473

Elston CW and Ellis IO (1991) Pathological prognostic factors in breast cancer. The value of histological grade in breast cancer: experience from a large study with long-term follow-up. *Histopathology* **19**(5): 403–410

Hietanen P, Blomqvist C, Wasenius V-M, Niskanen E, Franssila K and Nordling S (1995) Do DNA ploidy and S-phase fraction in primary tumour predict the response to chemotherapy in metastatic breast cancer? *Br J Cancer* **71**: 1029–1032

Krajewski S, Bodrug S, Gascoyne R, Berean K, Krajewska M and Reed JC (1994*a*) Immunohistochemical analysis of mcl-1 and bcl-2 proteins in normal and neoplastic lymph nodes. *Am J Pathol* **145**: 515–525

Krajewski S, Krajewska M, Shabaik A, Miyashita T, Wang H-G and Reed JC (1994*b*) Immunohistochemical determination of in vivo distribution of bax, a dominant inhibitor of bcl-2. *Am J Pathol* **145**: 1323–1333

Krajewski S, Blomqvist C, Franssila K, Krajewska M, Wasenius V-M, Niskanen E, Nordling S and Reed JC (1995) Reduced expression of proapoptotic gene Bax is associated with poor response rates in combination chemotherapy and short survival in women with metastatic breast adenocarcinoma. *Cancer Res* **55**: 4471–4478

Mansour EG, Ravdin PM and Dressler L (1994) Prognostic factors in early breast carcinoma. *Cancer* **74**: 381–400

Niskanen E, Franssila K, Blomqvist C, Hietanen P and Wasenius V-M (1997) Predictive value of c-erbB-2, p53, cathepsin-D and histology of the primary tumour in metastatic breast cancer. *Br J Cancer* **76**: 917–922

Reed JC (1994) Bcl-2 and the regulation of programmed cell death. *J Cell Biol* **124**(1–2): 1–6

Remvikos Y, Beuzeboc P, Zajdela A, Voillemot N, Magdelenat H and Pouillart P (1989) Correlation of pre-treatment proliferative activity of breast cancer with response to cytotoxic chemotherapy. *J Natl Cancer Inst* **1**: 1383–1387

Ro S, Sahin A, Ro J, Fritsche H, Hortobagyi G and Blick M (1990) Immunohistochemical analysis of P-glycoprotein expression correlated with chemotherapy resistance in locally advanced breast cancer. *Hum Pathol* **21**: 787–791

Sjöström J and Blomqvist C (1996) Predictive factors for response to cytotoxic treatment in advanced breast cancer. A review. *Acta Oncol* **35**(Suppl. 5): 84–90

Verrelle P, Meissonnier F, Fonck Y, Feillel V, Dionet C, Kwiatkowski F, Plagne R and Chassagne J (1991) Clinical relevance of immunohistochemical detection of multidrug resistance P-glycoprotein in breast carcinoma. *J Natl Cancer Inst* **83**: 111–116

© Cancer Research Campaign 1998